# Infiltrating macrophages increase RCC epithelial mesenchymal transition (EMT) and stem cell-like populations *via* AKT and mTOR signaling

**DOI:** 10.18632/oncotarget.9873

**Published:** 2016-06-07

**Authors:** Zhao Yang, Hongjun Xie, Dalin He, Lei Li

**Affiliations:** ^1^ Sex Hormone Research Center, Department of Urology, The First Affiliated Hospital, Xi'an Jiaotong University, Xi'an 710061, China

**Keywords:** RCC, macrophage, EMT, stem cell, AKT

## Abstract

Infiltrating macrophages are a key component of inflammation during tumorigenesis and progression. However, the role of macrophages in renal cell carcinoma (RCC), especially in the stage of RCC malignant progression, is still unclear. Here, we found the macrophages could be recruited more easily into RCC tissues than the surrounding non-tumor tissues. *In vitro* co-culture system also confirmed RCC cells had a better capacity to recruit macrophages *via* CXCL8 signaling than normal renal epithelial cells. The consequences of recruiting more macrophages may then increase RCC cells invasion abilities. Mechanism dissection revealed that infiltrating macrophages could function through induction of epithelial-mesenchymal transition and increased cancer stem cell-like populations *via* activation of AKT/mTOR signal, and then led to increasing RCC cells invasion. The orthotopically xenografted mouse model with RCC cells and macrophages also confirmed that infiltrating macrophages could increase RCC cells progression *via* AKT/mTOR signal. Together, our results reveal a new mechanism that macrophages in the RCC tumor microenvironment could increase RCC metastasis *via* activation of the AKT/mTOR signals. Targeting this newly identified signaling may help us to better inhibit RCC metastasis.

## INTRODUCTION

Renal cell carcinoma (RCC) accounts for approximately 3% of adult malignancies and 90–95% of kidney neoplasms [1]. Worldwide incidence and mortality rates are reported to be steadily rising at a rate of approximately 2–3% per decade [2]. Surgery remains the only effective treatment for localized RCC. Metastatic disease is usually resistant to radiation- and chemo-therapy, and immunotherapy shows limited response rates of 15% to 20% [3]. Although the development of protein tyrosine kinase inhibitor therapy opens a new door for advanced RCC patients, the overall survival is still limited for patients with selective pathological types [4]. Searching for a new and better therapy *via* new targets for RCC is still urgently needed.

Recent reports indicated that tumor-associated immune cells have been involved in the RCC initiation and progression, which could be an essential factor for the prediction of the outcome of tumor patients [5, 6]. Several immune cells in the RCC tumor microenvironment (TME), including macrophages, T cells, natural killer (NK) cells, dendritic cells (DCs) and neutrophils, might be recruited into RCC to exert their differential influences on tumor proliferation and invasion [7].

Macrophages are often viewed as double agents in the TME since their functional plasticity enables them to switch to a phenotype that is either for or against tumor development and progression dependent on M1 (classical) or M2 (alternative) activation [8]. It has been reported that the presence of extensive tumor associated macrophages (TAMs) infiltration into RCC TME contributes to cancer progression and metastasis by stimulating angiogenesis [9], and tumor growth, cellular migration and invasion [10]. Moreover, TAMs are involved in RCC cancer cells resistance to targeted agents [11]. Pharmacological depletion of macrophages in different mouse tumor models significantly reduced tumor angiogenesis and progression, suggesting that TAMs could be a potential target for RCC progression [12]. However, the detailed roles of macrophages in RCC invasion still remain unclear.

Here we found infiltrating macrophages could enhance the RCC invasion ability *via* increasing epithelial mesenchymal transition (EMT) and stem cell-like populations. The mechanism dissection identified that infiltrating macrophages mediated RCC invasion *via* the activation of AKT/mTOR signal. Targeting this newly identified signaling could be a potential strategy to better inhibit RCC metastasis.

## RESULTS

### Infiltrating macrophages are correlated with RCC development and progression

**Figure 1:**
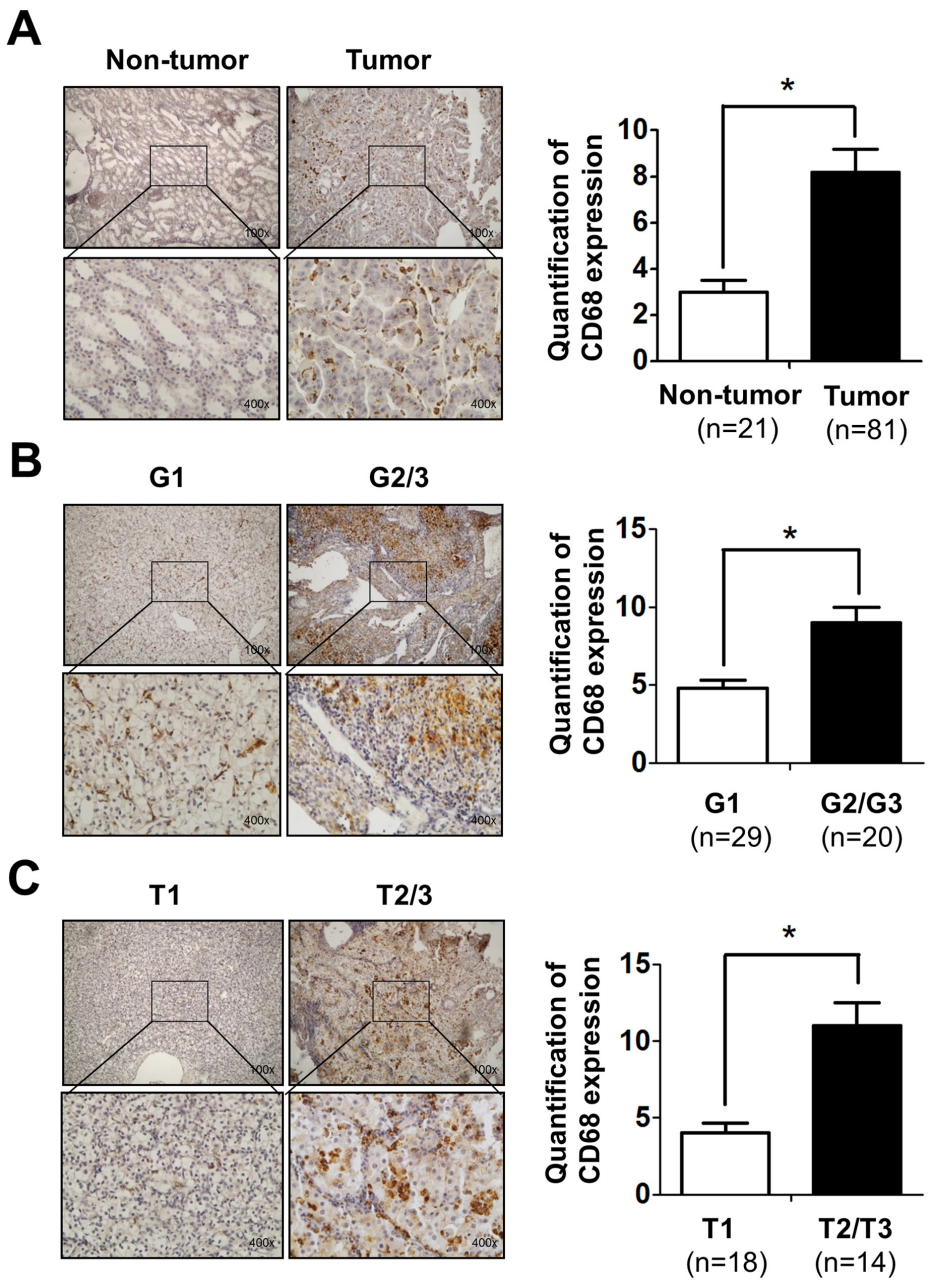
Infiltrating macrophages is positively related to RCC patients' tumor stage and grade **A.** IHC staining for CD68 as a marker of macrophages in RCC and non-tumor tissues (left panel). Quantitative data of CD68 positive cells in RCC and non-tumor kidney tissues (right panel). Upper: 100X; lower: 400X. * p < 0.05. **B.** IHC staining shows the CD68-positive cells in G1-G2/G3 grade of RCC patients (left panel). The right panel shows the quantification data. Upper: 100X; lower: 400X. * p < 0.05. **C.** IHC staining to show the CD68-positive cells in T1-T2/T3 stage of RCC patients (left panel). The right panel shows the quantification data. Upper: 100X; lower: 400X. * p < 0.05.

To investigate the potential linkage or impacts of infiltrating macrophages, the major immune cells existing in the kidney tumor microenvironment, in RCC progression, we applied IHC with anti-CD68 antibody, a specific marker of macrophages in human RCC and surrounding non-tumor tissues. The results revealed that the numbers of CD68-positive macrophages was significantly increased in RCC tissues compared to those in surrounding non-tumor tissues (Figure 1A). Importantly, we found more CD68-positive macrophages are linked to higher grade (G2/3) and stage (T2/3) RCC than the low grade (G1) and stage (T1) patients (Figure 1B-1C). Taken together, results from human clinical RCC samples indicated that infiltrating macrophages are positively correlated with the RCC development/progression.

### RCC cells have better capacity than normal renal epithelial cells to recruit macrophages

**Figure 2:**
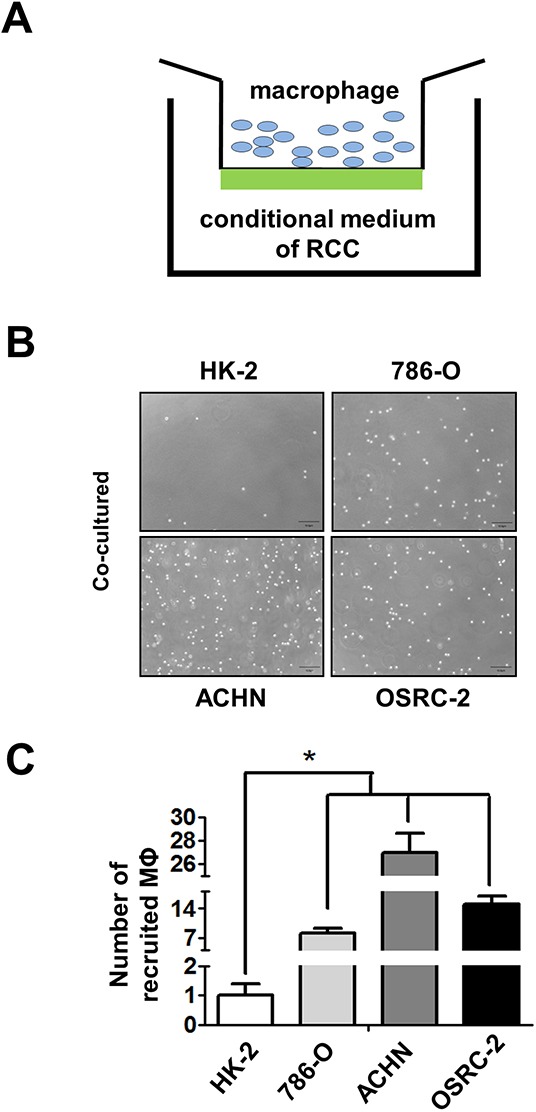
RCC cells have better capacity than normal renal epithelial cells to recruit macrophages **A.** The cartoon to show the recruitment assay. THP-1 cells were placed in upper chamber, and lower chamber was filled with CM of RCC cells. **B.** The recruitment assay in normal kidney (HK-2) and three RCC cell lines (786-O, ACHN, and OSRC-2). THP-1 cells passed through the membrane and recruited into the lower chamber as shown. **C.** The quantitative data of recruited macropahges (MФ) THP-1 cells by normal kidney and RCC cells were counted by haemocytometer. Results were presented as the mean±SEM. Statistical analysis was done by two-tailed Student's t test, * p < 0.05.

Next, to confirm human clinical sample surveys results above, we tested the THP-1 and RAW264.7 monocytes/macrophages migration ability towards RCC cells *vs.* renal proximal tubular epithelial cells (see illustration in Figure 2A), THP-1 cells were seeded on the upper chamber and the lower chamber was filled with the conditioned media (CM) of co-cultured THP-1 with/without RCC or HK2 cells. The M2 markers CD206 and CD163 expression of THP-1 cells were identified before the experiments (Figure S1A-S1B). After 20 h incubation, migrated cells (into bottom chamber) were counted and the results showed CM from co-culturing THP-1 or RAW264.7 cells with RCC cells including 786-O, ACHN and OSRC-2, had better capacity to recruit THP-1 or RAW264.7 cells into the bottom chamber than the normal HK2 cells (Figure 2B, S2A and S3A). The quantitative data also showed that CM of co-cultured THP-1 or RAW264.7 with RCC cells have better recruitment macrophages capabilities than the CM of co-cultured with normal HK2 cells (Figure 2C, S2B and S3B). There is no significant difference in TAMs recruitment between co-cultured and non-co-cultured CM (Figure S2C). Our results suggested that RCC cells have better capacity than normal renal epithelial cells to recruit macrophages.

### RCC could recruit more macrophages *via* increasing CXCL8 cytokine expression

**Figure 3:**
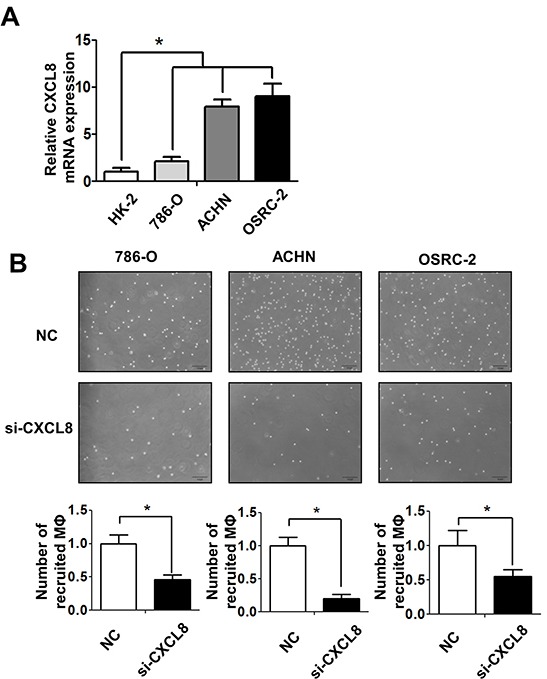
RCC cells recruit macrophages *via* secreting the CXCL8 cytokine **A.** The expressions of CXCL8 in normal kidney (HK-2) and different RCC cell lines (786-O, ACHN and OSRC-2) were assayed by Q-PCR, * p < 0.05. **B.** The recruitment capabilities of THP-1 macrophages (MФ) were counted by haemocytometer after knocking down CXCL8 by siRNA in 786-O, ACHN, OSRC-2 cells (lower panels) and their quantification data was shown at lower panel, * p < 0.05.

To dissect the mechanisms why RCC cells had a better capacity than normal renal epithelial cells to recruit macrophages, we then performed Q-PCR array assays to examine the potential candidate cytokines/chemokines that are related to the macrophages recruitment (Supplementay Figure S4A). The results showed a higher CXCL8 expression in co-cultured (Figure 3A) or non-co-cultured (Supplementay Figure S2D) 786-O, ACHN and OSRC-2 cells than normal HK2 cells. Importantly, using interruption assay with CXCL8-siRNA, we found knocking-down CXCL8 in RCC cells led to partial suppression of THP-1 cells migration (Figure 3B upper panels and Figure S3B). The quantitative data confirmed that knocking-down CXCL8 in RCC cells could block the macrophage recruitment capabilities significantly (Figure 3B lower panels).

In addition, RCC cells expressed more CXCL8 than THP-1 cells (Supplementay Figure S5A), and knocking down CXCL8 by siRNA did not inhibit the growth and invasion of 786-O and ACHN cells (Supplementay Figure S5B-S5D).

Taken together, studies from Figure 3A and 3B suggested that RCC cells could recruit more macrophages from its TMEs *via* altering the expression of CXCL8.

### Infiltrating macrophages increased RCC cells invasion capabilities

**Figure 4:**
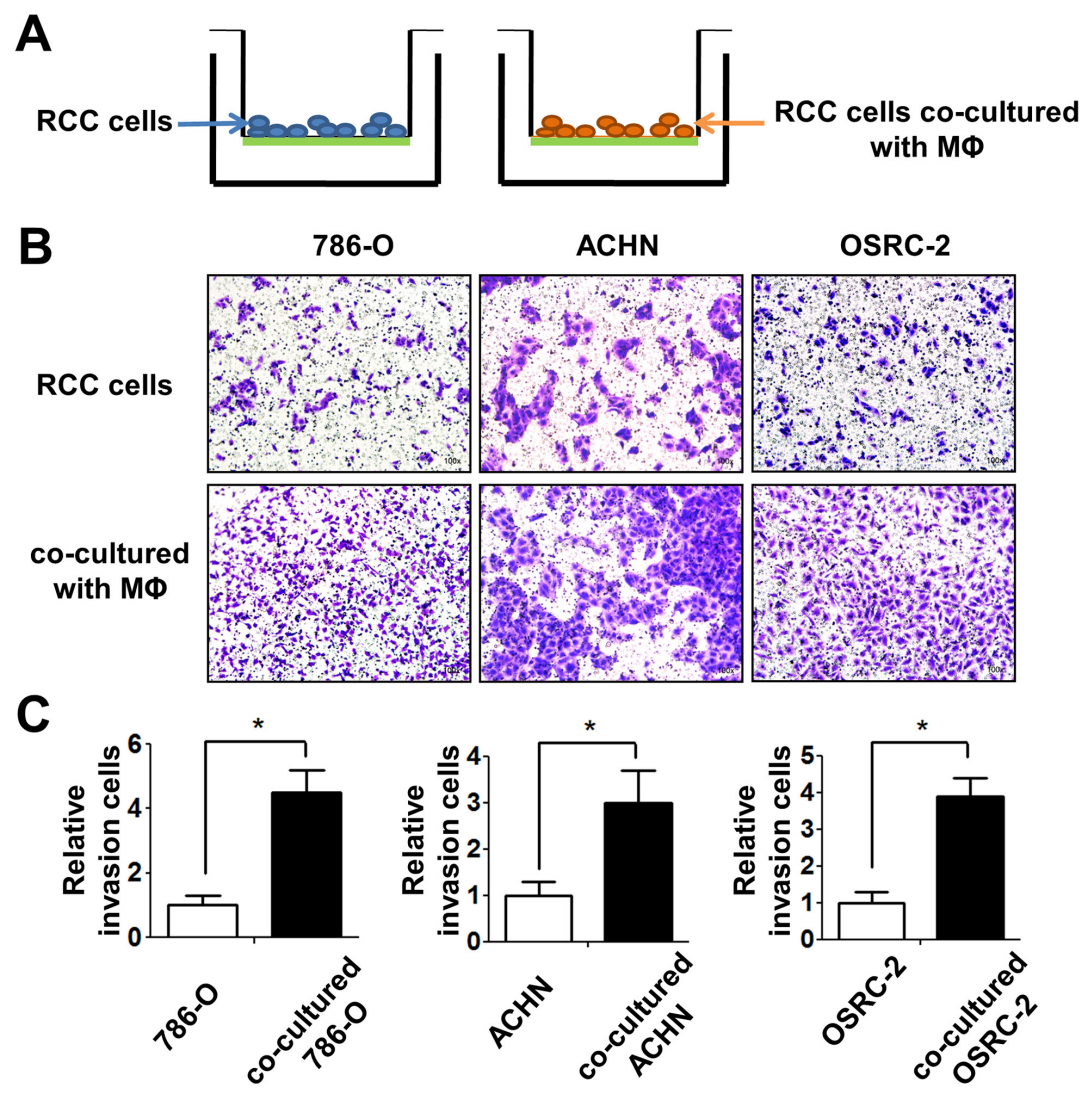
Infiltrating macrophage cells can increase the invasion ability of RCC cells **A.** The cartoon illustrates the invasion assay. We culture RCC cells with/without THP-1 macrophages 72 (MФ) hours, then cells were collected and re-seeded in the 8 μm pore size insert wells pre-coated with matrigel to perform invasion assays. **B.** The invasion assay showed 786-O, ACHN, OSRC-2 cells after co-culturing with THP-1 macrophages compared with control RCC cells. **C.** The quantitative results of RCC cells invasion were presented as the mean±SEM. Statistical analysis was done by two-tailed Student's t test, * p < 0.05.

To examine the potential consequences after recruiting macrophages into RCC tumor microenvironments, we focused on its potential effects on RCC cell invasion. We tested the invasion ability after culturing RCC 786-O, ACHN and OSRC-2 cells with/without TPH-1 and RAW264.7 macrophages, using 0.4um membrane inserts as described in materials and methods (Figure 4A). The results showed 786-O, ACHN and OSRC-2 cells co-cultured with macrophages had higher invasion ability than control RCC cells (Figure 4B and Supplementay Figure S3C-S3D left panel). The quantitative data confirmed that infiltrating macrophages increased the RCC cell invasion ability significantly. (Figure 4C and Supplementay Figure S3C-S3D right panel).

### Infiltrating macrophages increased RCC cells invasion *via* alteration of RCC EMT and increasing stem cell-like population

To dissect the potential molecular mechanisms why infiltrating macrophages increased RCC cells invasion, we focused on EMT-involved cancer stem/progenitor cells (CSCs) populations since several studies indicated that altering the EMT-involved stem/progenitor cell population might play key roles in promoting the cancer metastasis [13].

**Figure 5:**
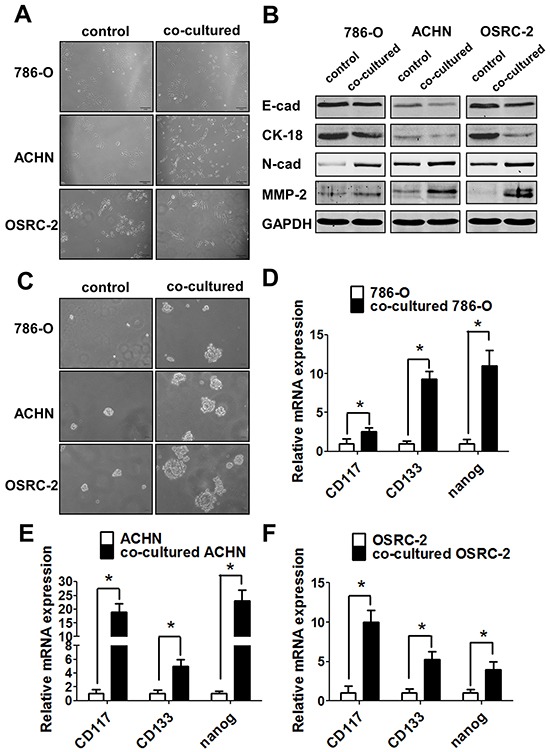
Infiltrating macrophage cells can promote RCC invasion *via* EMT alteration and increasing RCC stem cell-like population **A.** After 72h co-cultured, THP-1 macrophages could induce the morphology of 786-O, ACHN and OSRC-2 cells to change into long spindle-shape and disperse to each other compared to control cells. **B.** Western blot shows the E-cadherin, N-cadherin, CK-18 and MMP-2 expressions in RCC cells co-cultured with THP-1 macrophages compared with the control RCC cells. **C.** The sphere formation assay after co-culturing with THP-1 macrophages in 786-O, ACHN and OSRC-2 cells compared with control RCC cells. **D-F.** Q-PCR to detect the stem cell markers CD117, CD133 and nanog expressions in 786-O (D), ACHN (E) and OSRC-2 (F) cells with/without co-culturing with THP-1 macrophages. Statistical analyses were done by two-tailed Student's t test, * p < 0.05.

As shown in Figure 5A, after co-culturing with macrophages for 3 days, the morphology of 786-O, ACHN and OSRC-2 cells became long spindle-shaped, which fits the mesenchymal cell phenotype. Importantly, results from western blot analysis also showed the increased expressions of mesenchymal markers N-cadherin and MMP2 and decreased expressions of epithelial surface markers E-cadherin and Cytokeratin 18 (CK18) in RCC cells co-cultured with macrophages (Figure 5B). The results suggested that infiltrating macrophages could promote the EMT in the RCC cells.

To link the modulation of EMT to the alteration of the CSCs-like population in RCC after co-culturing with the macrophages, we then examined the CSCs feature with tumor sphere formation assay to confirm the above findings. The results indicated that RCC cells had a better performance in sphere formation capabilities showing much larger size of co-cultured 786-O, ACHN, and OSRC-2 cells as compared to the control cells (Figure 5C). To further confirm the alteration of CSCs following the co-culture with the macrophages, we then assayed the key markers of CSCs, including CD117, Nanog and CD133. The results suggested that infiltrating macrophages could increase the expression of CD117, Nanog and CD133 in co-cultured 786-O (Figure 5D), ACHN (Figure 5E), and OSRC-2 (Figure 5F) cells significantly.

Together, the results from Figure 5A-5F showed that infiltrating macrophages could increase RCC cells invasion capabilities mainly *via* altering RCC cells EMT and increasing stem cell-like population.

### Infiltrating macrophages increased RCC EMT and stem cell-like population *via* altering AKT/mTOR signals

**Figure 6:**
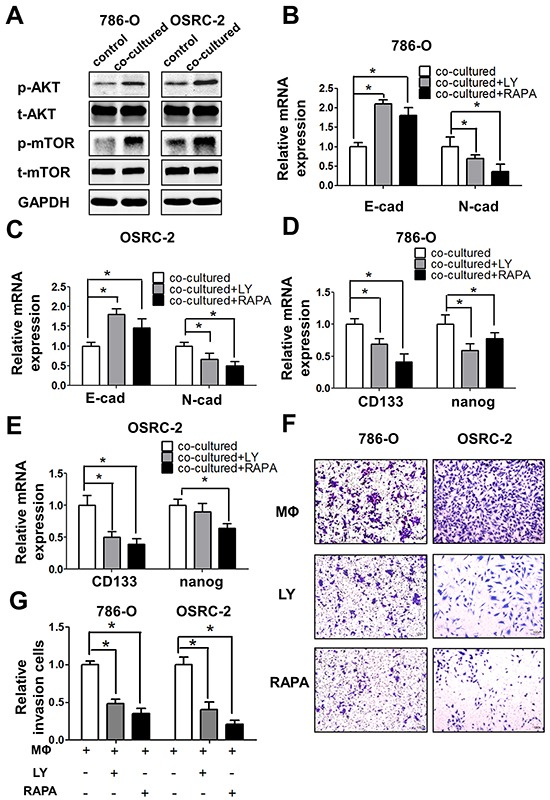
Infiltrating macrophage cells can promote RCC cells EMT and alter RCC stem cell-like population *via* AKT/mTOR signal **A.** Western blot shows the expression of p-AKT and p-mTOR in 786-O and OSRC-2 cells after co-culturing with THP-1 macrophages for 72 h compared with control RCC cells. **B** and **C.** Q-PCR shows the expression of E-cadherin (E-cad) and N-cadherin (N-cad) expression in co-cultured 786-O (B) and OSRC-2 (C) cells after adding PI3-K/AKT inhibitor LY294002 (LY) and mTOR inhibitor Rapamycin (RAPA) * p < 0.05. **D** and **E.** Q-PCR shows the expressions of CD133 and nanog in co-cultured 786-O (D) and OSRC-2 (E) cells after adding PI3-K/AKT inhibitor LY294002 (LY) and mTOR inhibitor Rapamycin (RAPA) * p < 0.05. **F** and **G.** Representative and quantitative invasion data of 786-O and OSRC-2 cells cultured with/without THP-1 macrophages by adding LY294002 (LY) and Rapamycin (RAPA). **H** and **I.** Quantitative invasion data of 786-O and OSRC-2 cells by blocking AKT or mTOR signals. Results were presented as the mean±SEM. Statistical analysis was done by two-tailed Student's t test, * p < 0.05.

To further dissect the mechanisms how macrophage cells promote RCC cells EMT and increase CSCs population, we focused on AKT/mTOR signals since recent studies indicated that AKT pathway is a central mechanism controlling EMT/CSC features, despite its definite effects on cancer cell proliferation and survival [14, 15]. The results suggest that infiltrating macrophages increased both the p-AKT, p-mTOR and Rictor signals in RCC cells compared to control cells (Figure 6A and Supplementay Figure S6A).

Importantly, using the interruption assay with the mTOR and AKT inhibitors rapamycin and LY294002, we showed that rapamycin could degrade p-AKT, as well as p-mTOR in 786-O cells (Supplementay Figure S6B), and could also partially block/reverse macrophages-enhanced EMT markers in co-cultured 786-O (Figure 6B) and OSRC-2 cells (Figure 6C). Similarly, The CSCs markers CD133 and nanog expression were also inhibited upon the blocking of AKT/mTOR signals with inhibitors in co-cultured RCC 786-O (Figure 6D) and OSRC-2 cells (Figure 6E). The consequence of interruption of AKT/mTOR signals showed the significant decrease of the invasion abilities in both co-cultured RCC cells *in vitro* invasion results (Figure 6F) and the quantitative data (Figure 6G).

Together, results from Figure 5A-5F and Figure 6A-6G suggested that infiltrating macrophages might enhance RCC cell invasion *via* altering the AKT/mTOR signaling, and then lead to the increasing of EMT and CSCs population.

### Infiltrating macrophages promote RCC tumorigensis *in vivo* mouse model

**Figure 7:**
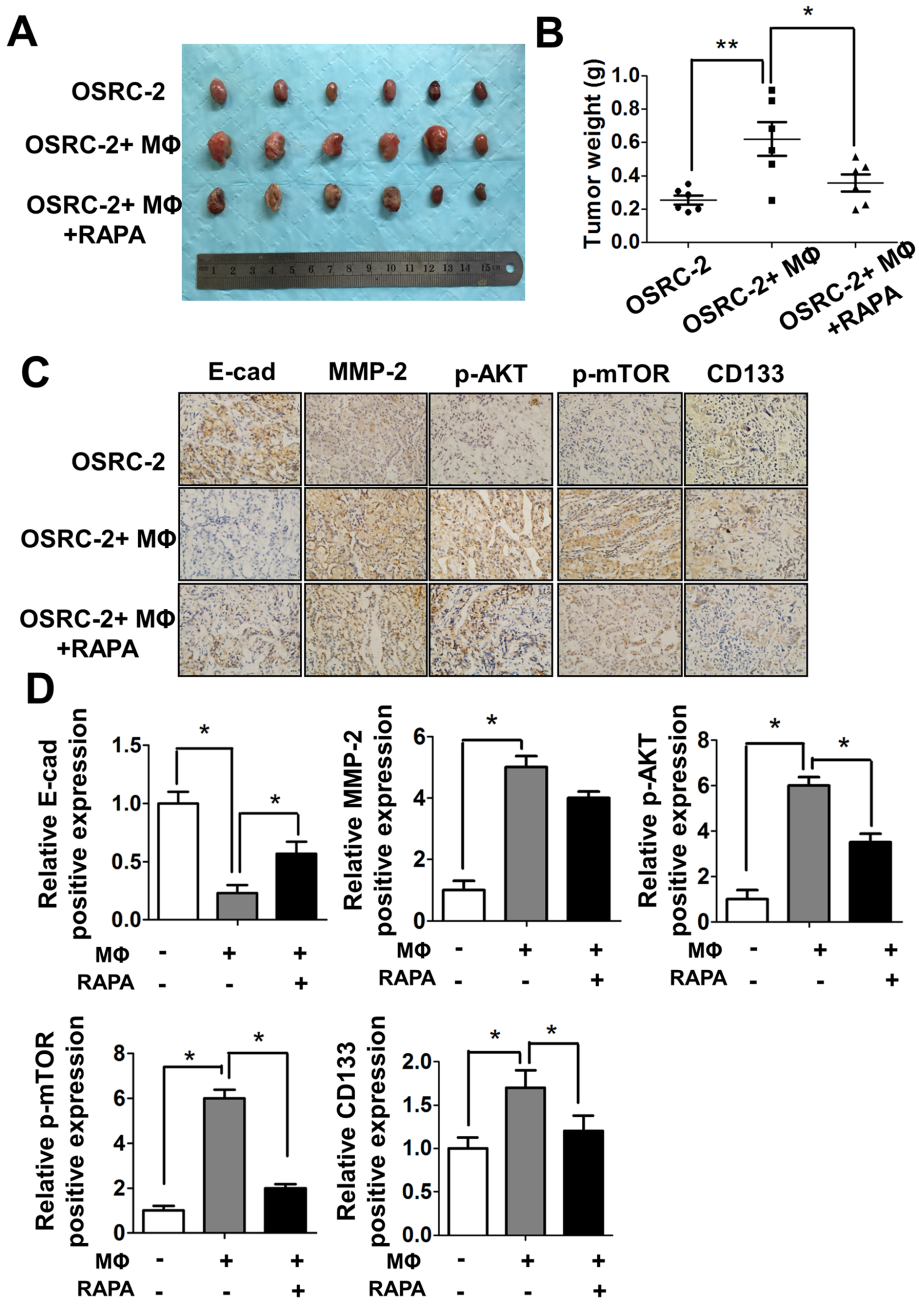
Macrophages can promote RCC tumorigenesis *in vivo* **A.** Tumor size were shown after xenografting 1×10^6^ OSRC-2 cells with/without mixed 1×10^5^ human THP-1 macrophages (MФ) for six weeks, mice were treated with Rapamycin (RAPA) 2mg/kg/day by intraperitoneal (IP). **B.** Tumor weights were presented as the mean±SEM. Statistical analysis was done by two-tailed Student's t test, * p < 0.05, ** p < 0.01. **C.** Representative IHC staining to shows the E-Cadherin, MMP-2, p-AKT, p-mTOR and CD133 expression in mice tumor tissues. **D.** Quantitative data of E-Cadherin, MMP-2, p-AKT, p-mTOR and CD133 expression presented as the mean±SEM. Statistical analysis was done by two-tailed Student's t test, * p < 0.05.

To demonstrate all above *in vitro* cell lines results in the *in vivo* animal models, we orthotopically xenografted OSRC-2 cells into the renal capsule of nude mice with or without human macrophage cells for six weeks, The results indicated that OSRC-2 cells co-injected with macrophage cells could increase the tumorigenesis abilities in mice significantly compared to OSRC-2 cells alone (Figure 7A and 7B). The *in vitro* data confirmed that the co-cultured RCC 786-O and ACHN cells have better growth ability than the control cells (Supplementay Figure S7A-S7B). Blocking mTOR signaling with specific inhibitor rapamycin could severely reverse the tumorigenesis abilities of cells co-injection with macrophages (Figure 7A and 7B). Moreover, the *in vivo* IHC staining (Figure 7C) and quantitative data (Figure 7D) also confirmed the *in vitro* results that macrophages could enhance RCC EMT and CSCs related protein MMP-2, p-AKT, p-mTOR, CD133 expression and decrease E-cadherin expression. The EMT and CSCs related protein expression could be reversed by treating the mice with mTOR specific inhibitor rapamycin.

Together, results from Figure 6A-6G and Figure 7A-7D conclude that macrophage cells are involved in the tumorigenesis of RCC, and targeting m-TOR signaling could be a potential candidate strategy to treat RCC.

## DISCUSSION

Renal cell carcinoma (RCC) has been considered highly resistant to both chemotherapy and radiation therapy, which is why other therapeutic approaches have been investigated. The tumor microenvironment with selective inflammatory immune cells, including T cells, NK, dendritic cells (DCs), and macrophages, also play an important role in RCC progression [16]. TAMs are one type of these immune cells that could be recruited into various tumor tissues to impact the tumor progression *via* influencing the angiogenesis [17], development of drug resistance and tumor progression [18]. However, whether TAMs can impact the RCC cells EMT, increase CSCs populations, and what are the detailed mechanisms involved remains unclear.

Our results provide the first *in vitro* and *in vivo* evidence that infiltrating macrophages could promote the RCC development and invasion *via* increasing RCC EMT alteration and CSCs populations. TAMs can adapt to the hypoxia status that characterizes RCC, resulting in an enhanced expression of pro-angiogenic genes, such as HIF/VEGF and IL-8, thus significantly supporting tumor growth and invasion [19]. It also reported that TNF-α-induced inhibition of glycogen synthase kinase-3β (GSK-3β) activity was also involved in RCC metastasis [20]. In addition, an extensive cross-talk between the Notch and TGF-β signaling pathways in RCC that is associated with aggressiveness has been reported [21]. Recent reports also found the infiltrating macrophages in the peri-tumoral compartment could be a novel independent prognostic marker for RCC [22, 23]. In agreement with previous results, our finding that recruited macrophages could enhance RCC invasion provides strong *in vitro* and *in vivo* evidence to support the macrophage roles in the RCC progression *via* alteration of RCC EMT.

CSCs are a subpopulation of oncogenic cells that have an ability to self-renew and the potential for generating heterogeneous malignant progenies [24, 25]. CSCs are thought to be responsible for cancer initiation, progression, and metastasis. Furthermore, they can acquire resistance to chemotherapy and oxidative stress. Induction of EMT-like features, including alterations in morphology toward a fibroblast-like appearance and an increase in the expression of EMT-related molecules, such as Slug and Snail, also increased expression of the CSC-related markers, Sox2 and Oct4. Cancer cells passing through EMT exhibit stem cell-like traits and CSCs can also acquire mesenchymal-like characteristics [26–28]. Our mechanism dissection found recruited macrophages might function through modulation of AKT signaling to enhance RCC EMT and stem-like cell population, which is in agreement with an early study showing that macrophages could promote CSCs in breast cancer [29]. Moreover, here we identified that AKT/mTOR signaling activation in the tumor microenvironments played the key roles of RCC tumorigenesis and progression.

In RCC, the overall genetic alteration rate of the representative PI3K/AKT pathway panel components was 27.7%. Most of the components were identified with genetic alterations, including GNB2L1 amplification (6%) and mTOR mutations (6%), in a largely mutually exclusive manner [30]. mTOR mutations are highly clustered in small regions in RCC, conferring mTOR hyperactivation [31]. The PI3k/Akt/mTOR protein cascade is one of the three major signaling pathways associated with receptor tyrosine kinases that have been identified in cancer cells [32]. Inhibition of PI3K isoforms, AKT, mammalian targets of rapamycin (mTOR), and other components in the pathway are being actively pursued for targeted cancer therapy [33]. It has been demonstrated that the VHL/HIF and PI3K/AKT pathways cross-talk extensively in a large signaling network, contributing to RCC [34, 35]. This is consistent with our observation that AKT/mTOR signaling is not only involved in RCC cells development, but also contributed to the TAMs mediated RCC progression. Targeting mTOR signaling might represent a new therapy to better suppress RCC progression.

In conclusion, we identified infiltrating macrophages as a new key player to promote the RCC tumorigenesis and progression and targeting mTOR with rapamycin to suppress CSCs and EMT alteration may help us to develop a new therapy with better efficacy to fight RCC.

## MATERIALS AND METHODS

### Cell culture and co-culture experiments

HK2 (human renal proximal tubular epithelial cell line), 786-O, ACHN and OSRC-2 cells (human RCC cell lines) were maintained in DMEM/F12 medium (Invitrogen, Carlsbad, CA, USA) with 10% fetal bovine serum (Invitrogen, Carlsbad, CA, USA). THP-1 cells were maintained in RPMI-1640 medium with 10% fetal bovine serum. All cell lines were purchased from ATCC (American type culture collection, ATCC. USA). THP-1 cells were differentiated into macrophages by phorbol 12-myristate 13-acetate (PMA, Sigma-Aldrich, Paso Robles, CA) stimulation before study [36, 37]. For co-culture experiments, 3 × 10^4^ THP-1 cells were seeded in the upper insert (0.4 μm) of six-well transwell plates (Corning Inc., Corning, NY) which contains RCC cells or HK2 cells (3×10^5^ cells) in the lower chamber with RPMI-1640 with 10% FBS or serum free medium for 24h, and the co-cultured CM was collected for further experiments.

### Antibodies and chemicals

GAPDH (6c5), E-cadherin (H-108), Vimentin (V9) and MMP-2 (H-76) antibodies were purchased from Santa Cruz Biotechnology (PasoRobles, CA). Phospho-Akt (Ser473 D9E) and Akt (11E7) antibodies were purchased from Cell Signaling Technology Company (Boston, MA), Phospho-mTOR (ab109268) and mTOR (ab32028) antibodies were from Abcam Company (San Diego, CA). CD68 (M087601-2) antibody were purchased from DAKO (Carpinteria, CA). Rapamycin was from Cell Signaling Technology Company (Boston, MA). Crystal violet was from Fishers Scientific Company (Grand Island, NY). Anti mouse/rabbit second antibody for Western Blot and Lipofectamine 2000 transfection reagent were purchased from Life Technologies Company (Grand Island, NY).

### Macrophage recruitment assay

The macrophage recruitment was assayed as previously described [38]. In brief, The RCC and THP-1 cells were co-cultured in serum free medium for 24 h, then the CM were collected, plated into the lower chamber of 24-well transwell plates with 5 μm pore polycarbonate membrane insert. 1×10^5^ of THP-1 cells were plated into the upper chamber for macrophage migration assay. After 20 hrs, the cells migrated into the lower chamber were collected and centrifuged, and counted using a haemocytometer.

### Cell invasion assay

*In vitro* cell invasion assay was performed using 24-well (8 μm pores) transwell inserts [39]. Briefly, the upper chambers of the transwells were precoated with diluted gelatinous protein mixture (Dilution ratio: 1:4. Matrigel, BD Biosciences, Sparks, USA). Then 5×10^4^ RCC cells were plated onto the upper chamber after co-culturing with macrophages or CM for 72 hrs. After 24 hrs, invaded cells were stained with 0.1% crystal violet, and positively stained cells were counted. The cell numbers were averaged from counting five random fields.

### Rna extraction and quantitative real-time PCR analysis

Total RNA from cells was isolated with Trizol reagent (Life Technologies, Rockville, MD, USA) and 1 μg of total RNA was subjected to reverse transcription using the PrimeScript™ RT reagent kit (Takara, Dalian, China). Quantitative real-time PCR was conducted using a CFX96 real-time PCR system (Bio-Rad, Hercules, CA, USA) with SYBR Green PCR Master Mix (Takara, Dalian, China) to determine the mRNA expression level of a gene of interest. Expression levels were normalized to the expression of GAPDH RNA.

### Western blot analysis

After treatment, cells were washed with cold PBS three times, then the total cellular protein lysates were prepared with RIPA buffer [50 mM Tris pH 8.0, 150 mM NaCl, 0.1% SDS, 1% NP40 and 0.5% sodium deoxycholate] containing proteinase inhibitors [1% Cocktail and 1 mM PMSF, both from Sigma, (St Louis, MO, USA)]. Individual samples (30–35 μg of protein) were prepared for electrophoresis run on 8–10% SDS-PAGE and transferred to Nitrocellulose Membranes. After blocking membranes with 5% skim milk at room temperature for 1 h, they were incubated with appropriate dilutions (1:1000) of specific primary antibodies, and then the blots were incubated with HRP-conjugated secondary antibodies and visualized using Odyssey Detection System (Licor, Rockford, IL, USA).

### *In vivo* tumorigenesis studies

The use of animals and the experimental protocol were approved by the Institutional Animal Care and Use Committee of Xi'an Jiaotong University (permit NO. SCXK2014-0155, 5 March 2014). 6 male 6-8 weeks old nude mice were injected with 1×10^6^ OSRC-2 cells (mixed with matrigel, 1:1v/v) and 12 other mice were co-injected with OSRC-2 cells (1×10^6^) and macrophages (1×10^5^) into renal capsule. Rapamycin (RAPA) was used in co-injected with OSRC-2 cells and macrophage cells 6 mice at the dosage of 2 mg/kg per day by intraperitoneal (IP) injection after xenographed for 2 weeks. The mice were scarified and tumor sizes were measured at 6 weeks. The tumors tissues were further examined by H&E and IHC staining.

### Immunohistochemistry

The EnVisionTM System (DAKO, Carpinteria, CA, USA) was used for IHC staining according to the protocol recommended by the manufacturer. Tumor sections were deparaffinized, rehydrated and subjected to heat-induced antigen retrieval. Endogenous peroxidase and alkaline phosphatase activity were blocked with 3% H_2_O_2_ in methanol for 20 min. The slides were then incubated overnight at 4°C with primary antibodies. After washing three times, slides were incubated with Envision secondary antibody for 30 min at room temperature. Then, signals were detected by diaminobenzidine (DAB) buffer followed by hematoxylin counterstaining. Slides were viewed and photographed using an Olympus BX51 Microscope (Olympus, Tokyo, Japan).

### Statistics

All statistical analysis were carried out with SPSS 16.0 (SPSS Inc, Chicago, IL). The data values were presented as the mean±SEM. ANOVA test was used for analyzing the discrepancy of the three or more groups comparition. The Student's t-test was used to detect any statistically significant difference between two groups. A p-value less than 0.05 was considered to be statistically significant.

## SUPPLEMENTARY FIGURES


