# Supplementary material for: Infiltrating macrophages increase RCC epithelial mesenchymal transition (EMT) and stem cell-like populations via AKT and mTOR signaling
Source: Oncotarget. 2016 Jun 7;7(28):44478–91. doi: 10.18632/oncotarget.9873 (PMC5190112; doi:10.18632/oncotarget.9873)
Supplement: Supplementary file 1 [file oncotarget-07-44478-s001.pdf]

Infiltrating macrophages increase RCC epithelial mesenchymal transition (EMT) and stem cell-like populations *via* AKT and mTOR signaling

SUPPLEMENTARY FIGURES

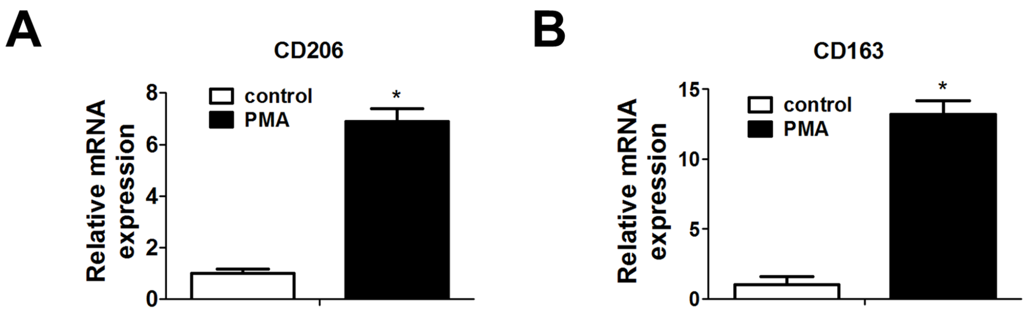

Supplementary Figure S1: PMA treatment can induce up-regulation of M2 macrophage markers. A. Expression of CD206 after PMA treatment * $p < 0.05$. **B.** Expression of CD163 after PMA treatment * $p < 0.05$.

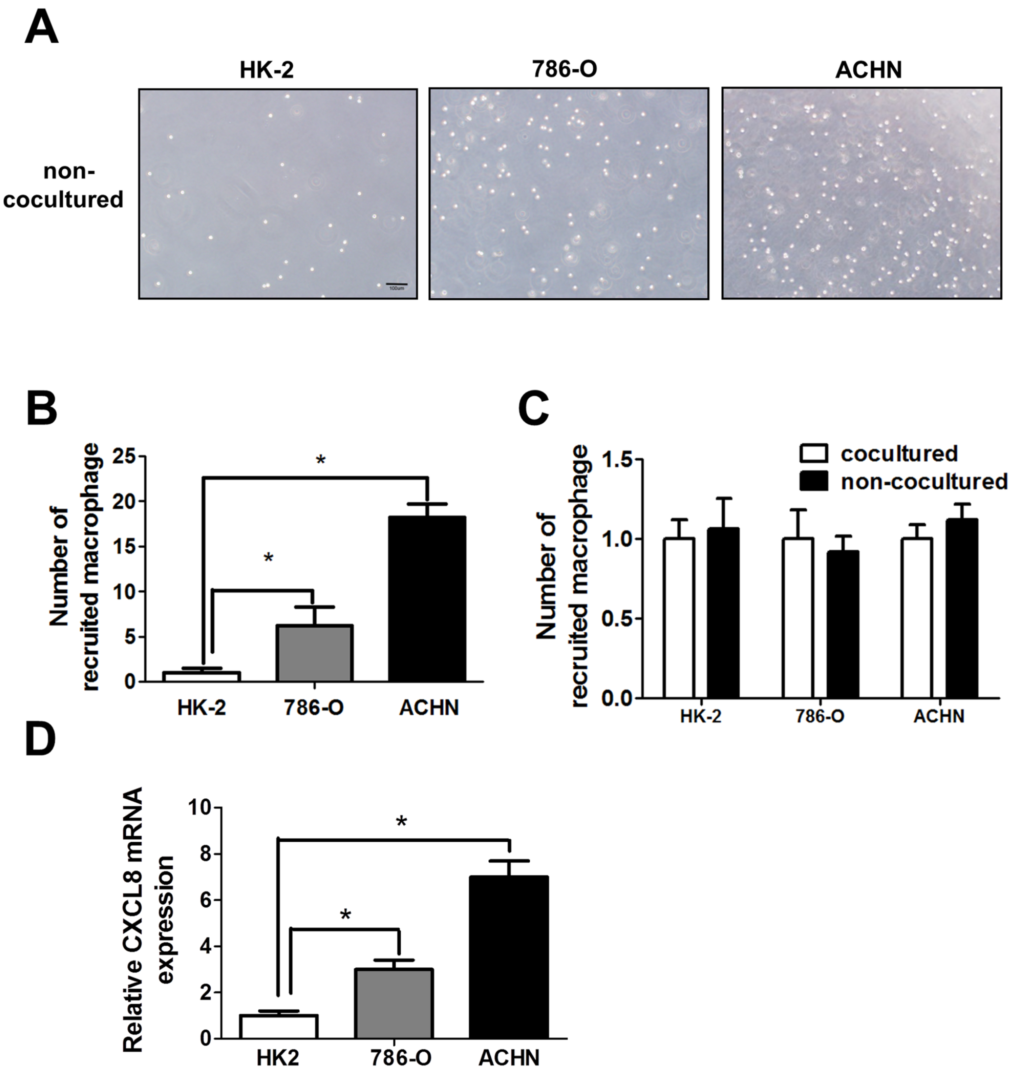

Supplementary Figure S2: The recruitment ability of RCC alone conditional medium compared with co-cultured with macrophage conditional medium. **A.** Macrophage recruitment assays by RCC alone conditional medium in normal HK-2 cells and RCC 786-O and ACHN cells. **B.** The quantitative data of conditional medium of RCC and HK-2 cells alone to recruit macrophages * $p < 0.05$. **C.** The quantitative data compared RCC alone conditional medium with co-cultured conditional medium to attract macrophages. **D.** q-PCR assays compared the expression of CXCL8 in RCC 786-O, ACHN cells and normal HK-2 cells without co-culturing with macrophages * $p < 0.05$.

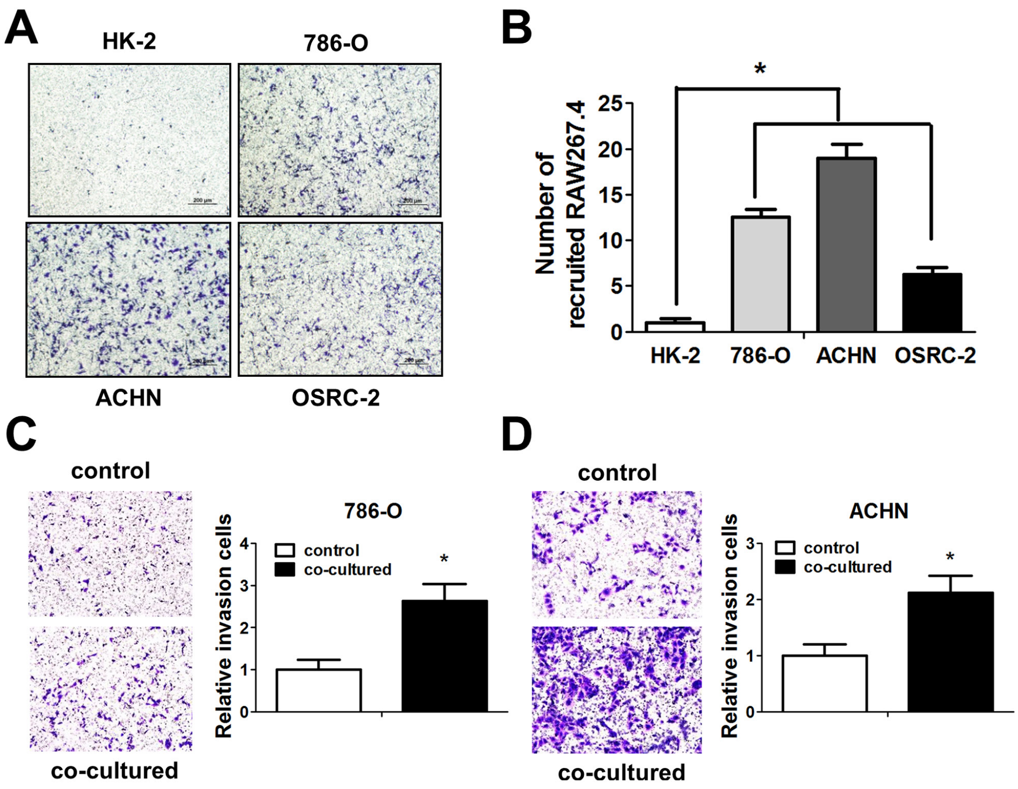

Supplementary Figure S3: Infiltrating RAW267.4 macrophage cells promote RCC invasion ability. A. The recruitment assays in normal kidney (HK-2) and three RCC cell lines (786-O, ACHN, and OSRC-2). **B.** The quantitative data of recruitment assays of 786-O, ACHN, OSRC-2 and HK-2 cells. Results were presented as the mean ± SEM. Statistical analysis was done by two-tailed Student's t test, * $p < 0.05$. **C.** Invasion ability of 786-O cells after co-culturing with RAW267.4 cells * $p < 0.05$. **D.** Invasion ability of ACHN cells after co-culturing with RAW267.4 cells * $p < 0.05$.

A

Target Genes	786-O/HK2	ACHN/HK2	OSRC-2/HK2
CD62L	20.412	0.627	2.343
CSF1	9.096	1.391	21.242
EGF	1.982	0.672	1.462
VEGFA	1.094	2.182	0.0769
FGF	0.781	1.65	1.013
CCL2	0.032	0.015	0.295
CCl4	1.872	0.165	0.672
CCL5	N/A	0.421	1.194
CCL5	N/A	0.034	N/A
CCL8	0.502	N/A	1.254
CXCL2	0.018	3.192	0.272
CXCL3	0.29	1.878	1.313
CXCL4	0.021	N/A	0.067
CXCL5	0.072	2.003	3.176
CXCL6	1.767	0.936	N/A
CXCL7	1.238	0.312	5.097
CXCL8	2.435	8.161	9.023
CXCL9	N/A	1.573	0.039
CXCL11	0.651	0.123	1.197
CX3CL1	0.015	1.892	0.173

B

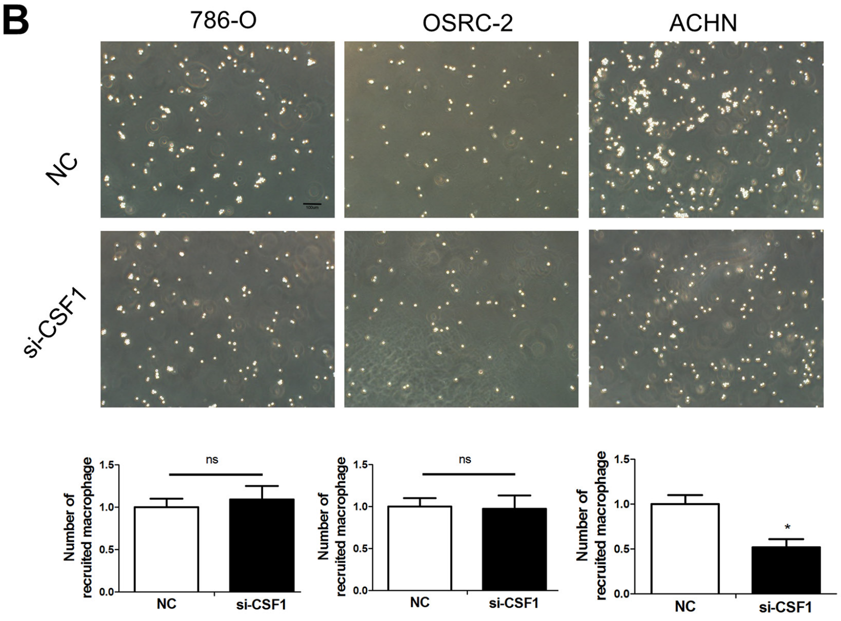

Supplementary Figure S4: The recruitment related gene transcription profile in 786-O/HK2, ACHN/HK2 and OSRC-2/HK2 cells using Q-PCR array assay. A. The transcriptional levels of CXCL8 were dramatically increased in RCC cells compared with normal epithelial cells. **B.** Interruption assay with CSF1-siRNA in ACH, 786-O and OSRC-2 cells * $p < 0.05$.

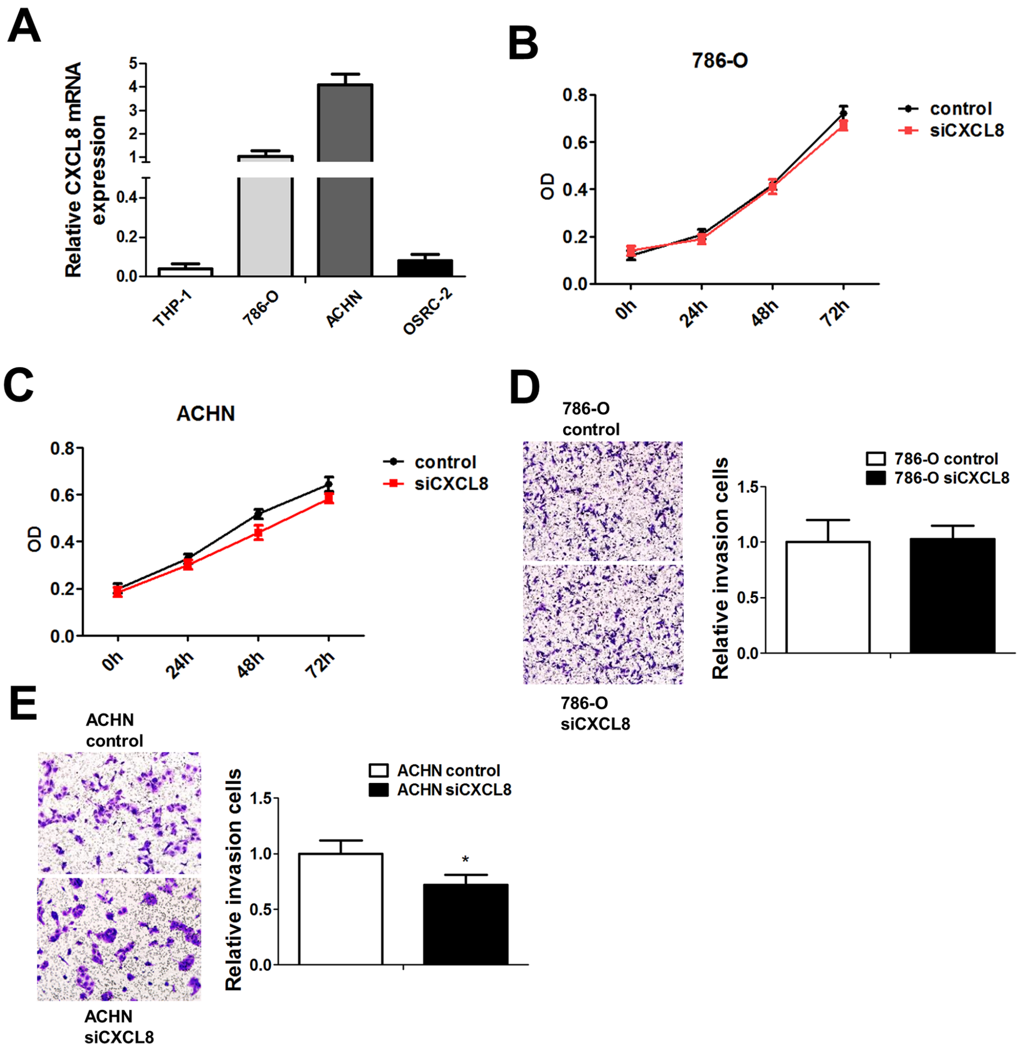

Supplementary Figure S5: CXCL8 is abundant in RCC and limited effect to RCC. A. CXCL8 expression compared macrophage with RCC 786-O, ACHN and OSRC-2 cells. **B and C.** MTT assays to test the growth ability after knocking down CXCL8. **D and E.** The invasion assay showed 786-O and ACHN cells after knocking down CXCL8 compared with control RCC cells * $p < 0.05$.

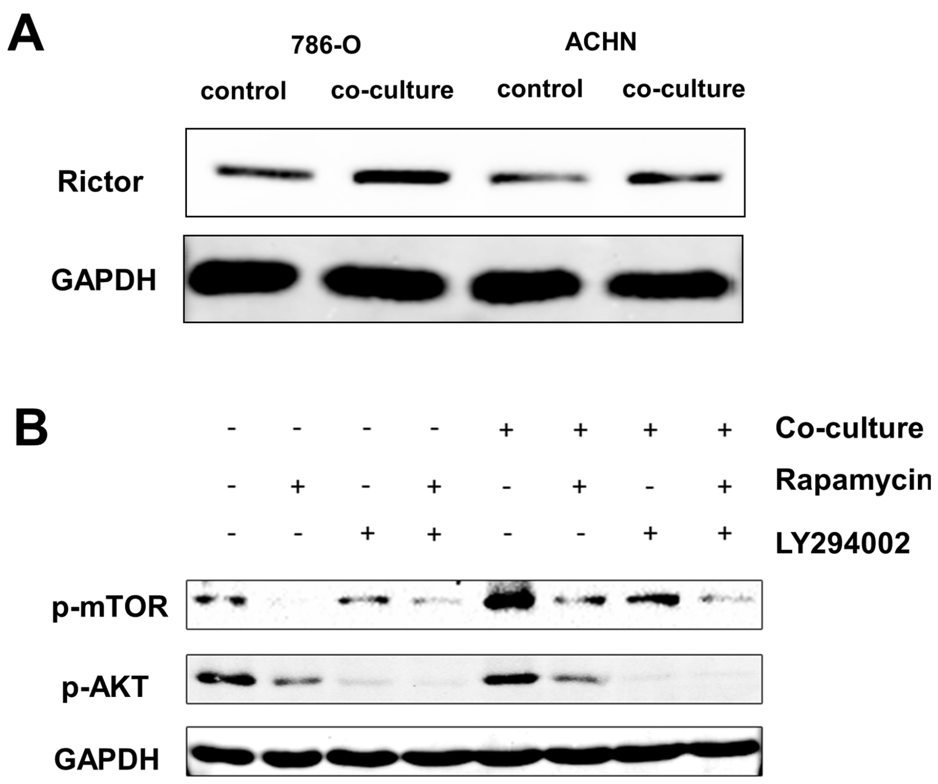

Supplementary Figure S6: Infiltrating macrophage cells can induce up-regulation of Rictor expression in RCC. A. Rictor expression in 786-O and ACHN cells after co-culturing with macrophage. **B.** western blot shows the protein level of p-AKT and p-mTOR after adding LY294002 and Rapamycin.

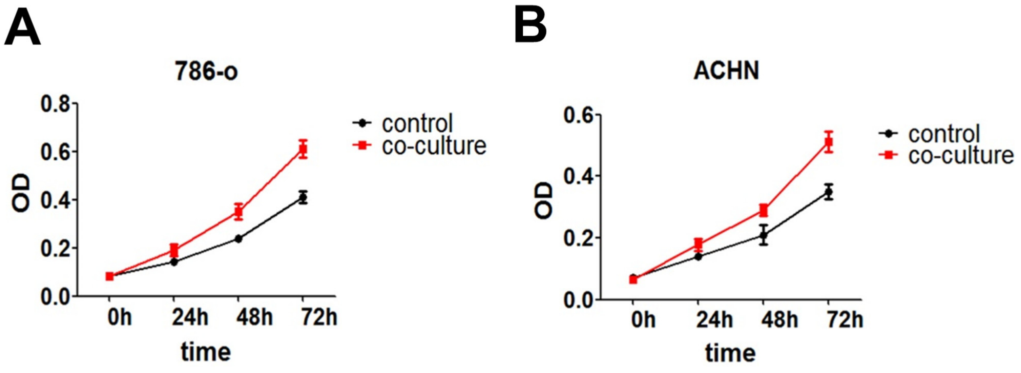

Supplementary Figure S7: Infiltrating macrophage cells can promote RCC proliferation. A. MTT assay shows 786-O cells after co-culturing with macrophages compared with control RCC cells. **B.** MTT assay shows ACHN cells after co-culturing with macrophages compared with control RCC cells.
